# An Improved PointPillars-Based Dual-LiDAR Method for Aircraft Relative Pose Estimation in Towbarless Towing Vehicles †

**DOI:** 10.3390/s26185780

**Published:** 2026-09-11

**Authors:** Yu Zhu, Falian Li, Hongfeng Yan, Liang Cui

**Affiliations:** 1 Chinese Academy of Agricultural Mechanization Sciences, Beijing 100083, China; yuzhu_zy@163.com (Y.Z.); gonfyuou@protonmail.com (F.L.); cuiliang_2008@126.com (L.C.); 2 Beijing Jinlun Kuntian Special Machine Co., Ltd., Beijing 100083, China; 3 National Key Laboratory of Agricultural Equipment Technology, Beijing 100083, China

**Keywords:** 3D object detection, PointPillars, towbarless towing vehicle (TLTV), LiDAR, self-attention mechanism, point-cloud density feature

## Abstract

To address the oversteering risk during aircraft ground towing with a towbarless towing vehicle, this study proposes a dual-LiDAR point-cloud detection and pose estimation method for aircraft rear-wheel targets. First, a complementary dual-LiDAR acquisition strategy is adopted to reduce rear-wheel point-cloud occlusion caused by the aircraft nose landing gear and towing mechanism. Second, considering the small size and distinctive local geometry of rear-wheel targets, vertical density enhanced encoding and a lightweight CNN-Transformer BEV backbone are introduced into the PointPillars framework. The vertical density enhanced encoding explicitly describes the normalized height-wise distribution of valid points within each pillar, thereby improving the representation of cylindrical wheel structures. The CNN-Transformer BEV backbone incorporates a window-based self-attention Transformer module into deep features to strengthen local contextual modeling in the BEV space. Based on the detected coordinates of the left and right rear wheels, the aircraft fuselage pose is then estimated in combination with the TLTV coordinate system. In three-seed experiments on the fixed validation split, the Full model achieves an mAP@0.5 of 0.8788±0.0161, which is 8.50 percentage points higher than the original PointPillars baseline. The model contains 4.1069 M parameters and runs at 38.0732 FPS. The towing-angle estimation error remains within the allowable engineering range. These results show that the task-specific adaptations improve rear-wheel detection while retaining a compact model and real-time processing capability.

## 1. Introduction

Intelligent vehicles have become a representative direction in the development of mobile equipment. Such systems commonly follow a modular technical route that includes multi-sensor perception and fusion, object detection and tracking, target state or pose estimation, motion prediction, decision planning, and assisted control. Among these modules, LiDAR point clouds are widely used for autonomous-driving 3D object detection because they provide direct three-dimensional geometric information [1,2]. Airport ground support equipment is undergoing a similar transition from mechanization and automation toward intelligent operation. The towbarless towing vehicle (TLTV) is an important component of modern airport ground support systems. It is mainly used for aircraft pushback, transfer, and towing while the aircraft engines are shut down. During operation, towing force is transmitted through clamping or lifting the nose landing gear, which creates a direct coupling among vehicle motion, the clamping-center position, and the nose-wheel.

The nose landing gear is a key structure for aircraft ground steering and towing-load transmission, and its maximum steering angle and allowable torque are strictly limited. FAA safety guidance indicates that improper towing procedures may damage the aircraft nose landing gear [3]. SAE recommended practices for the design and testing of towbarless towing vehicles also include operational safety, monitoring and warning, and nose-landing-gear oversteering protection as engineering constraints [4,5]. Studies on the structural strength of the nose landing gear under towing and taxiing conditions further show that towing places the nose landing gear in a complex load environment, making its structural safety closely related to aircraft ground-operation safety [6,7]. These sources show that oversteering protection is important for towing-operation safety and nose-landing-gear structural protection.

In aircraft relative-pose detection, the relative pose between the towing vehicle and the aircraft can be represented by the angle between their longitudinal axes. The longitudinal axis of the towing vehicle can be determined from the vehicle structure and sensor installation position, whereas the aircraft fuselage axis must be estimated from observable aircraft structures. Compared with the nose-landing-gear region, the rear wheels are located symmetrically on both sides of the fuselage and are spatially independent from the local motion of the clamping mechanism. They are therefore more suitable as detection targets for estimating the aircraft fuselage direction. By using the rear wheels as perception targets, the oversteering-detection problem can be transformed into a close-range, small-target LiDAR point-cloud detection problem, providing geometric input for subsequent pose calculation.

Towing and steering maneuvers introduce practical challenges for rear-wheel point-cloud perception. During aircraft ground towing, the clamping mechanism of the towbarless towing vehicle is directly coupled with the nose landing gear wheels. Consequently, the nose landing gear and adjacent fuselage structures may occlude the line of sight from a single LiDAR to the rear-wheel region, resulting in incomplete point-cloud observations. To reduce the influence of such occlusions on perception stability, this study employs a dual-LiDAR configuration to synchronously observe the rear-wheel region. When one LiDAR is affected by occlusion or insufficient sampling, the other LiDAR can provide a complementary viewpoint, thereby improving the spatial coverage of rear-wheel point clouds. Nevertheless, rear-wheel targets are small, and their point distributions in the vertical direction and along boundary regions remain sensitive to sampling density, incidence angle, and local occlusion. Therefore, a 3D detection model for this towing scenario should be specifically adapted to the sensor configuration, target geometry, and real-time deployment requirements rather than directly relying on a general-purpose detection framework.

PointPillars is a one-stage 3D object detection method for LiDAR point clouds. Its main idea is to divide a point cloud into vertical pillars and project pillar features into a BEV pseudo-image, which is then processed by a 2D convolutional network, achieving a favorable balance between speed and accuracy [8]. This structure avoids expensive 3D convolution and is suitable for engineering applications that require real-time perception in towing vehicles. However, the original PointPillars pillar encoder relies on point-wise feature aggregation and pooling, which can compress vertical-distribution details within each pillar. Its 2D convolutional backbone also mainly uses local convolution kernels, limiting its ability to use surrounding spatial relationships in sparse small-target scenarios. Aircraft rear wheels can be approximated as cylindrical objects with clear vertical structural characteristics, and they appear in pairs. Local convolution kernels alone cannot fully represent this spatial context. To address these issues, this study introduces two lightweight improvements into the main PointPillars framework: vertical density enhanced features in the pillar-encoding stage and a local window attention module in the high-level BEV backbone.

The main contributions of this study are as follows. A dual-LiDAR rear-wheel detection method is designed for the oversteering-protection requirements of towbarless towing vehicles, using aircraft rear wheels as key targets for aircraft pose estimation. A vertical density enhanced pillar feature encoding method is proposed to supplement height-distribution information for wheel targets without changing the main PointPillars detection paradigm. A lightweight CNN-Transformer BEV backbone is designed by embedding local window attention into the deep backbone to improve contextual modeling for small-target detection. Ablation experiments are conducted on a self-collected real LiDAR dataset for the original PointPillars, single-module variants, and the full model, and an oversteering-protection simulation is further completed in a simulated scenario.

This study substantially extends our preliminary conference work [9], which presented a rule-based point-cloud processing framework and preliminary towing-angle verification using the same dual-LiDAR experimental platform and registration method. The present study replaces the rule-based rear-wheel extraction stage with a learning-based 3D detection framework, introduces vertical-density-enhanced pillar encoding and a lightweight CNN–Transformer BEV backbone, expands the dataset to three front-to-rear wheel spacings, and provides systematic ablation, detection-accuracy, computational-efficiency, and repeated nine-angle verification experiments.

## 2. Related Work

### 2.1. LiDAR Point-Cloud 3D Object Detection

The key to LiDAR point-cloud 3D object detection is to extract features for object localization from sparse, unordered, and unevenly distributed 3D points and to predict the 3D position, size, and orientation of objects. Early deep-learning methods usually converted point clouds into regular representations so that convolutional neural networks could be used for feature extraction. MV3D encoded point clouds into bird’s-eye-view and front-view representations and fused them with image features, demonstrating the effectiveness of BEV representations for 3D proposal generation [10]. VoxelNet divided point clouds into 3D voxels and implemented end-to-end 3D object detection through voxel feature encoding [11]. Building on this idea, SECOND introduced sparse convolution to reduce the computational waste caused by empty voxels, improving the efficiency of voxel-based detection frameworks [12].

Point-based methods directly process raw point sets. PointNet provides a foundation for unordered point-set feature learning [13]. PointRCNN uses point-wise features for 3D proposal generation and detection [14], while 3DSSD further explores point-based one-stage detection to balance efficiency and fine-grained geometric preservation [15]. PV-RCNN combines point-level and voxel-level features, forming a compromise between regular encoding efficiency and precise localization [16]. CenterPoint represents 3D objects as center-point prediction problems in BEV space and is a representative method for autonomous-driving 3D detection [17].

Overall, existing LiDAR 3D detection methods have developed several technical routes, including point-based, voxel-based, BEV/pillar-based, and hybrid approaches. In airport TLTV scenarios, the target category is simple, wheel targets are small, and real-time performance is required. Model selection therefore depends not only on detection accuracy but also on training stability, inference efficiency, and engineering complexity. Compared with more complex point-voxel fusion frameworks, PointPillars provides a more suitable baseline for engineering verification through pillar encoding and a 2D BEV backbone.

Recent reviews of deep-learning-based point-cloud processing have emphasized that practical applications involve constraints beyond network architecture and benchmark accuracy, including large data volumes, diverse scene contents, varying point density, sensing modality, computational efficiency, and generalization across application environments [18]. These factors are relevant when a detector developed from general point-cloud methods is adapted to a specialized acquisition geometry and small-target distribution.

### 2.2. Pillar Representation and Attention-Based BEV Feature Modeling

Pillar representation divides a point cloud into vertical pillars and converts 3D detection into 2D feature extraction and object prediction on a BEV pseudo-image. PointPillars avoids the high computational cost of 3D convolution and shows clear efficiency advantages in real-time detection [8]. At the same time, studies such as PillarNet and PillarNeXt show that pillar-based models can still improve detection performance while maintaining efficiency through redesigned pillar feature encoding, neck structures, receptive fields, and training strategies [19,20]. Two-stage pillar feature encoding for PointPillars also indicates that the construction of pillar features remains a meaningful direction for improvement [21].

The high efficiency of pillar representation also comes with information compression. The original PointPillars pools point-wise features within the same pillar into a single pillar vector, so the height-wise point distribution within the pillar is mainly represented implicitly. BEV representation methods such as MV3D have used height, intensity, and density statistics to describe local point-cloud structure [10]. Studies such as SA-SSD and TANet also show, from the perspectives of structure awareness and attention enhancement, that target geometric details and key local features are practically useful for detection [22,23]. The vertical density enhanced encoding in this study follows the idea of height and density statistics and embeds it into the PointPillars pillar feature output, allowing vertical-distribution information and pillar features to jointly participate in subsequent BEV detection.

Attention mechanisms, graph structures, and object-relation modeling provide another form of supplementation for high-level BEV features. Transformer models use self-attention to model dependencies among feature locations [24], and Swin Transformer uses window attention to reduce the computational complexity of high-resolution visual features [25]. In 3D detection, VoTr and SST show that attention mechanisms can be used for sparse voxel or point-cloud feature modeling [26,27], while Group-Free 3D object detection further indicates that Transformers can learn contribution relationships between point features and object candidates [28]. Point-GNN applies graph structures to adjacency modeling in point-cloud object detection [29], and Relation Networks show in 2D object detection that spatial relationships among objects can supplement detection reasoning [30]. Although these studies do not directly address aircraft rear-wheel detection, they provide methodological inspiration for introducing local contextual modeling into high-level BEV features in this study.

Regarding oversteering protection, Existing oversteering-protection studies are mainly patent-based and focus on the nose-landing-gear steering angle, torque, turning-angle limit alarms, or TLTV control intervention [31,32,33,34]. These disclosures indicate that oversteering protection has received sustained attention in engineering practice; however, LiDAR-based rear-wheel object detection specifically designed for pose estimation in towbarless aircraft tractor oversteering protection remains relatively limited.

## 3. Methodology and System Architecture

This study proposes an aircraft pose estimation method based on a dual-LiDAR module for the oversteering-protection requirements of towbarless towing vehicle transfer operations. To reduce point-cloud occlusion of aircraft rear wheels by the nose landing gear during ground transfer, a complementary dual-LiDAR strategy is used to improve target point-cloud completeness. An improved 3D object detection model is then built on the PointPillars framework according to the target characteristics, with the model input consisting of point clouds acquired by the dual LiDARs and unified into a common coordinate system. Finally, the aircraft fuselage axis is estimated from the target positions and combined with the TLTV centerline to obtain the relative aircraft pose.

### 3.1. Dual-LiDAR Acquisition Module

**Figure 1:**
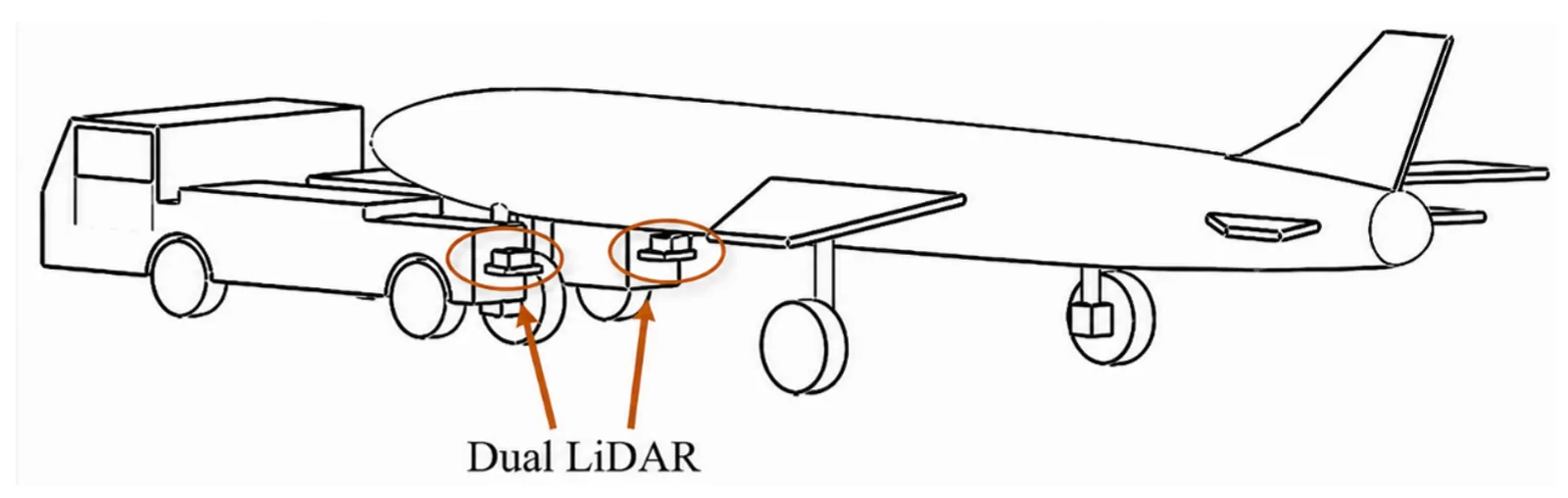
Schematic diagram of LiDAR mounting arrangement during aircraft towing.

The LiDAR mounting arrangement during aircraft towing is shown in Figure 1. Two LiDAR sensors are symmetrically mounted on both sides of the rear of the tow tractor, providing complementary point-cloud data when observations from one side are occluded or sparse. To fuse the point clouds acquired by the two LiDAR sensors, their individual coordinate systems are transformed into a vehicle coordinate system whose origin is located at the clamping center. The vehicle coordinate system follows the right-hand rule, with the positive *X*-axis pointing toward the rear of the tow tractor.

Point-cloud messages generated by the two LiDAR sensors were paired using an approximate timestamp synchronization method. The pairing was based on message-header timestamps generated using the ROS host clock. No hardware triggering, PTP, or GPS-based time synchronization was employed.

Let $F_{1}$ and $F_{2}$ denote the right-handed coordinate frames of LiDAR 1 and LiDAR 2, respectively, and let $F_{V}$ denote the vehicle frame whose origin is the TLTV clamping center. The source and target point sets are written as P1={pi1} and P2={pj2}, where the leading superscript identifies the frame in which a column vector is expressed. This study adopts a source-to-target coordinate-mapping convention with column vectors and left multiplication; no inverse or passive rotation is implied. The rigid transformation from $F_{1}$ to $F_{2}$ is

(1) T12=R12t120T1,R12∈SO(3),t12∈R3.

For the same physical point, Equation (1) gives

(2) p21=T12p11,p2=R12p1+t12.

The mounting geometry provides the initial estimate, after which Generalized ICP (GICP) refines the transformation [35]. Using the correspondence set K, the estimate is obtained from

(3) T^12=argminT12∈SE(3)∑(i,j)∈KrijTCj2+R12Ci1R1T2−1rij,

where the residual for a source–target pair is

(4) rij=pj2−R12pi1+t12.

Here, Ci1 and Cj2 are the local neighborhood covariance matrices of the source and target points in their native frames. The source covariance is rotated into $F_{2}$ in Equation (3). Point coordinates and translation components use meters, consistent with the point-cloud files.

Both scans are subsequently expressed in $F_{V}$. Let T2V be the fixed mounting transformation from LiDAR 2 to the vehicle frame and let $\tilde{p}={\left[p^{T},1\right]}^{T}$ denote a homogeneous point. The fused coordinates are

(5) p˜V=T2VT^12p˜1,p∈P1,T2Vp˜2,p∈P2.

To write a rotation matrix to a LiDAR configuration, the matrix is converted to roll–pitch–yaw angles using the standard right-handed ZYX factorization. Active rotations of column vectors are used, and the rightmost rotation is applied first:

(6) $$R=R_{z}\left(\psi \right)R_{y}\left(\theta \right)R_{x}\left(\phi \right),$$

where ϕ, θ, and ψ denote roll about the *x*-axis, pitch about the *y*-axis, and yaw about the *z*-axis, respectively. The elementary rotations are

(7) $$\begin{array}{cc} R_{x}\left(\phi \right) & =\left[\begin{array}{ccc} 1 & 0 & 0\\ 0 & \cos\phi & -\sin\phi \\ 0 & \sin\phi & \cos\phi \end{array}\right],\\ R_{y}\left(\theta \right) & =\left[\begin{array}{ccc} \cos\theta & 0 & \sin\theta \\ 0 & 1 & 0\\ -\sin\theta & 0 & \cos\theta \end{array}\right],\\ R_{z}\left(\psi \right) & =\left[\begin{array}{ccc} \cos\psi & -\sin\psi & 0\\ \sin\psi & \cos\psi & 0\\ 0 & 0 & 1 \end{array}\right]. \end{array}$$

With $c_{\phi }=\cos\phi$, $s_{\phi }=\sin\phi$, and analogous notation for θ and ψ, Equation (6) expands to

(8) $$R=\left[\begin{array}{ccc} c_{\psi }c_{\theta } & c_{\psi }s_{\theta }s_{\phi }-s_{\psi }c_{\phi } & c_{\psi }s_{\theta }c_{\phi }+s_{\psi }s_{\phi }\\ s_{\psi }c_{\theta } & s_{\psi }s_{\theta }s_{\phi }+c_{\psi }c_{\phi } & s_{\psi }s_{\theta }c_{\phi }-c_{\psi }s_{\phi }\\ -s_{\theta } & c_{\theta }s_{\phi } & c_{\theta }c_{\phi } \end{array}\right].$$

For the nonsingular case $\sqrt{R_{11}^{2}+R_{21}^{2}}>\varepsilon$, the principal ZYX angles are recovered using two-argument arctangent functions:

(9) $$\begin{array}{cc} \psi & =\mathrm{atan2}(R_{21},R_{11}),\\ \theta & =\mathrm{atan2}\left(-R_{31},\sqrt{R_{11}^{2}+R_{21}^{2}}\right),\\ \phi & =\mathrm{atan2}(R_{32},R_{33}). \end{array}$$

The angle ranges are ϕ,ψ∈(−π,π] and θ∈[−π/2,π/2]. When $\sqrt{R_{11}^{2}+R_{21}^{2}}\leq \varepsilon$, pitch is at a gimbal-lock configuration and roll and yaw cannot be identified independently. A deterministic export convention is used by setting ϕ=0 and computing

(10) $$\theta =\mathrm{atan2}\left(-R_{31},0\right),\;\psi =\mathrm{atan2}\left(-R_{12},R_{22}\right).$$

All trigonometric calculations use radians.

### 3.2. Original PointPillars Model

PointPillars is a one-stage 3D object detection framework for LiDAR point clouds. Its main idea is to compress a 3D point cloud into pillar-shaped voxels along the vertical direction and then convert them into a regular BEV pseudo-image. Compared with voxel-based detection methods that rely on 3D sparse convolution, PointPillars avoids expensive 3D convolution while maintaining high detection efficiency and 3D localization capability. It is therefore suitable for engineering scenarios such as real-time perception in towing vehicles.

**Figure 2:**
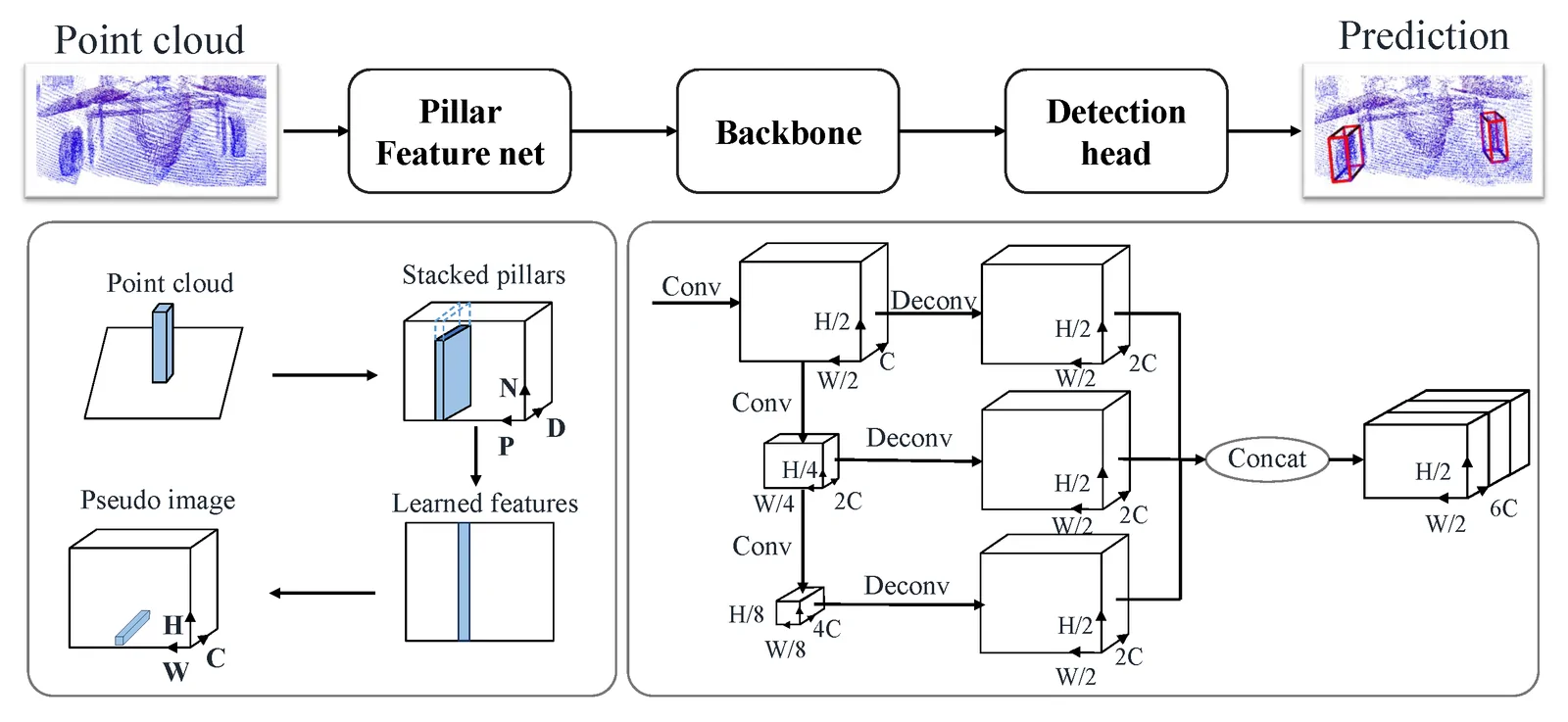
Framework of the original PointPillars model. Solid arrows indicate the data and feature flow, and red boxes in the prediction image denote the predicted 3D bounding boxes of the rear wheels.

The processing pipeline of the original PointPillars can be summarized as pillar partitioning, pillar feature encoding, pseudo-image generation, 2D backbone feature extraction, and 3D detection-head prediction, as shown in Figure 2. Given the raw point cloud, the model assigns points to different pillars according to their horizontal coordinates, and each pillar retains a fixed number of points. PillarFeatureNet then constructs enhanced features for each point, including the original coordinates, reflectance intensity, offsets relative to the point-cluster center, and offsets relative to the pillar center, and extracts point-wise representations through a shared MLP. To obtain fixed-length pillar-level features, the original model uses max pooling along the point dimension to aggregate the point features in each pillar into a compact pillar descriptor.

After features of non-empty pillars are obtained, PointPillarsScatter writes the sparse pillar features into their corresponding 2D grid positions to generate a BEV pseudo-image. This pseudo-image is then fed into a 2D convolutional backbone and a feature pyramid network for multi-scale feature extraction and fusion. The detection head predicts object classes, 3D bounding-box parameters, and orientation information on the BEV feature map using preset anchors. This pipeline is structurally simple, computationally efficient, and able to reuse mature 2D convolutional detection structures.

In Figure 2, *P* denotes the number of non-empty pillars in a point-cloud frame, *N* denotes the fixed maximum number of points retained in each pillar, and *D* is the dimension of the point-wise input feature. Thus, the stacked-pillar block represents *P* pillars, each containing at most *N* point records with *D* feature components. PillarFeatureNet converts each pillar into a *C*-dimensional feature vector, and the scatter operation arranges these features into a BEV pseudo-image of size C×H×W, where *C* is the number of feature channels and *H* and *W* are the height and width of the BEV grid, respectively. In the backbone, labels such as H/2, W/2, and 2C indicate spatial downsampling and channel expansion, while the deconvolution branches restore the multi-scale feature maps to the same spatial resolution before concatenation.

However, the original PointPillars still has two limitations for the task considered in this study. First, max pooling compresses the vertical distribution of points within each pillar, making it difficult for subsequent networks to directly use height-density differences in small cylindrical targets such as wheels. Second, the original 2D convolutional backbone mainly relies on local convolution kernels for feature extraction. As the target occupies a smaller region in deeper feature maps, a local receptive field alone is insufficient to describe the spatial relationship between wheel targets. Based on these two limitations, this study improves PointPillars in the feature encoding stage and the BEV backbone.

### 3.3. Overall Network Architecture

**Figure 3:**
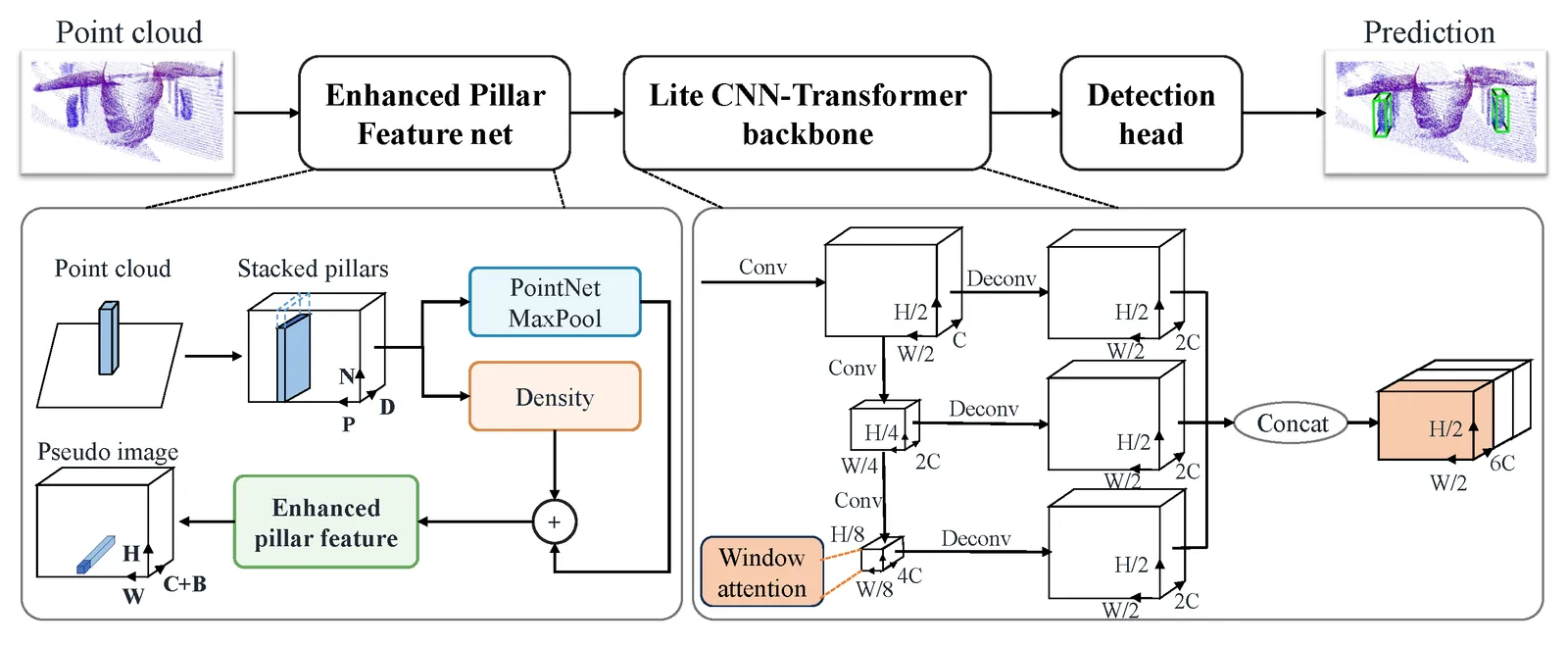
Framework of the improved PointPillars model. Solid arrows indicate the data and feature flow, dashed lines link the overview modules to their corresponding detailed structures, and green boxes in the prediction image denote the predicted 3D bounding boxes of the rear wheels.

The core algorithm follows an end-to-end pipeline of voxelization, feature encoding, multi-scale fusion, and object prediction. Point clouds within the detection range are divided into pillar-shaped voxels. The enhanced pillar feature encoder then extracts pillar features, which are converted into a BEV pseudo-image by a scatter operation, as shown in Figure 3. The pseudo-image is processed by the lightweight CNN-Transformer backbone and a multi-scale feature fusion module, and the 3D detection head outputs the 3D bounding boxes, class confidence scores, and orientation information of wheel targets. Compared with the original PointPillars, the proposed improvements are concentrated in two positions: vertical density distribution features are added in the pillar-encoding stage, and a local window attention mechanism is introduced into deep BEV backbone features. The former enhances the vertical structural representation of wheel targets, whereas the latter models contextual relationships around targets at a lower spatial resolution.

### 3.4. Vertical Density Enhanced Pillar Feature Encoding

The original PointPillars extracts point-wise features inside each pillar using a PointNet-like structure and compresses the point dimension through channel-wise max pooling. For pillar *p* containing $N_{p}$ valid points, let $h_{p,i}\in R^{C}$ denote the feature of its *i*th point. The pillar descriptor is

(11) $$f_{p}=\max_{i=1,\ldots ,N_{p}}\;h_{p,i},$$

The wheel targets considered in this study are typical small objects and have cylindrical characteristics. Max pooling can cause the vertical contour features of wheels to be lost, leaving the subsequent backbone without explicit vertical-contour information. Density differences among different height layers are weakened, which affects stable recognition of small targets.

**Figure 4:**
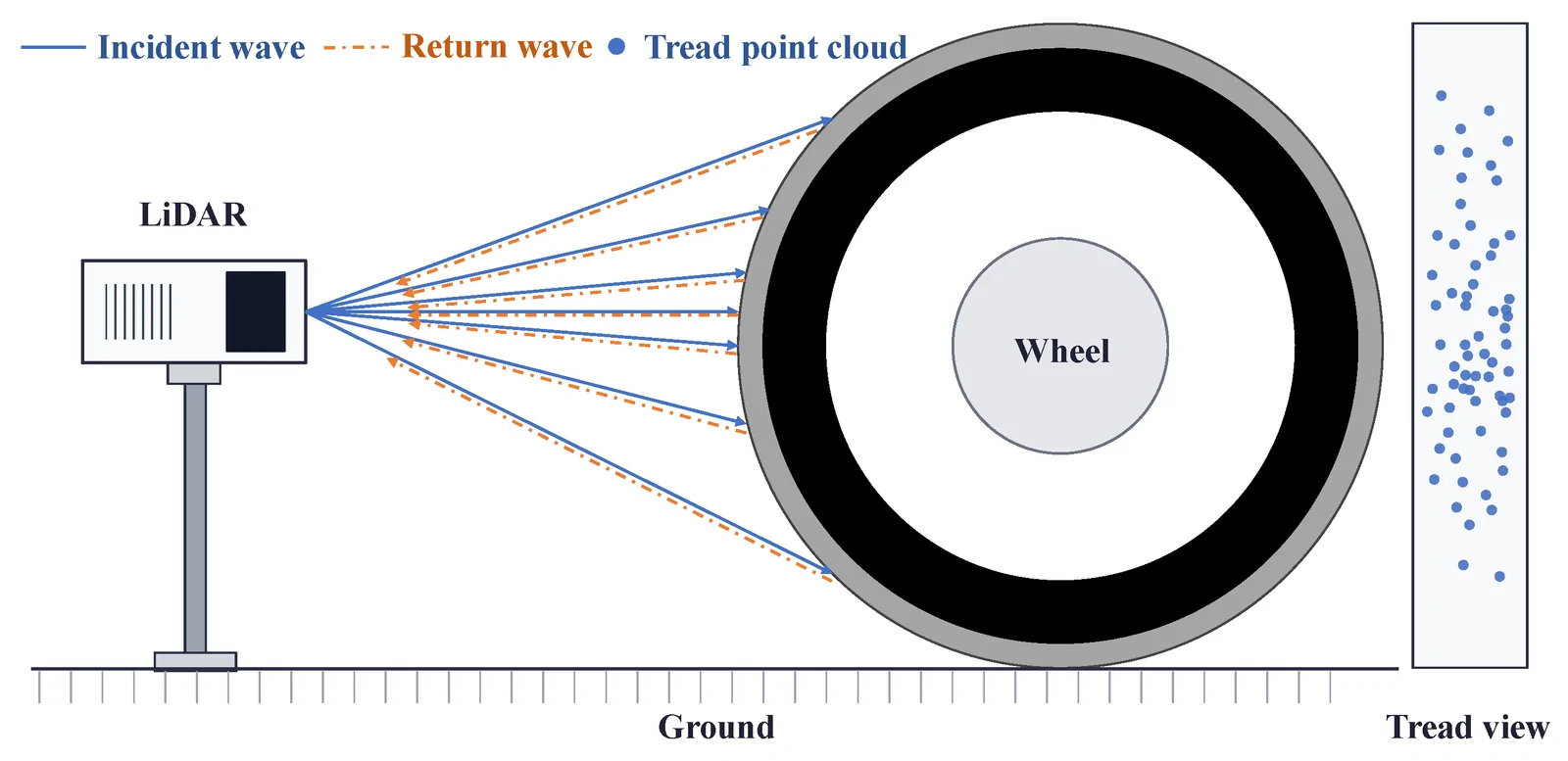
Schematic diagram of the LiDAR incidence-angle and tire.

To address the insufficient vertical information representation of standard PointPillars, this study proposes a vertical density enhanced pillar feature encoding method. Owing to the wheel geometry, viewpoint, self-occlusion, and sampling pattern, the returns from an aircraft wheel are not uniformly distributed along height, as illustrated schematically in Figure 4. The proposed descriptor therefore records the normalized height-wise point distribution within each pillar and fuses it with the standard pillar feature.

The configured point-cloud range $\left[z_{\min},z_{\max}\right)=\left[-0.5,3.0\right)$ m is uniformly divided into *B* intervals. The width of each interval is

(12) $$\Delta z=\frac{z_{\max}-z_{\min}}{B}.$$

The kth interval can be expressed as follows.

(13) $$B_{k}=\left[z_{\min}+k\Delta z,\;z_{\min}+\left(k+1\right)\Delta z\right),\;k=0,1,\ldots ,B-1.$$

For the *i*th point in pillar *p*, with height coordinate $z_{p,i}$, its height-layer index is

(14) $$b_{p,i}=\mathrm{clip}\left(\left\lfloor \frac{z_{p,i}-z_{\min}}{\Delta z}\right\rfloor ,0,B-1\right).$$

Here, clipping prevents boundary points from causing index overflow. Let $N_{p}$ be the number of valid points in pillar *p*. The normalized density of its *k*th height layer is

(15) $$\rho_{p,k}=\frac{1}{N_{p}}\sum_{i=1}^{N_{p}}I\left(b_{p,i}=k\right),\;k=0,1,\ldots ,B-1.$$

Here, I(·) is the indicator function. The resulting vector $\rho_{p}={\left[\rho_{p,0},\rho_{p,1},\ldots ,\rho_{p,B-1}\right]}^{T}$ satisfies $\sum_{k=0}^{B-1}\rho_{p,k}=1$ for a non-empty pillar. This descriptor does not replace the original PointPillars feature; it supplements it with normalized height-occupancy information.

(16) $$f_{p}^{\prime }=\mathrm{concat}\left(f_{p},\rho_{p}\right).$$

Here, $f_{p}\in R^{64}$ is the original PillarFeatureNet output, $\rho_{p}\in R^{8}$ is the vertical density vector (B=8), and $f_{p}^{\prime }\in R^{72}$ is the enhanced feature input to the scatter layer. These dimensions agree with the implemented model configuration.

### 3.5. Lightweight Hybrid Backbone Network

PointPillars uses the intermediate encoder PointPillarsScatter to convert pillar features into a pseudo-image. The original model uses a 2D CNN backbone to perform multi-layer downsampling and feature extraction on the pseudo-image. In shallow feature maps, CNN local kernels can capture low-level geometric information such as wheel edge curvature, local point-density changes, and ground-contact regions. As the CNN deepens, however, the feature-map resolution gradually decreases and semantic information becomes stronger. Local convolution alone is then insufficient to fully use the spatial context around the targets. In aircraft towing scenarios, wheel targets have relatively stable geometric constraints, and the targets also have a clear spatial reference relationship with the TLTV clamping center. Such contextual information can support detection under occlusion, long-distance observation, and sparse point-cloud conditions.

**Figure 5:**
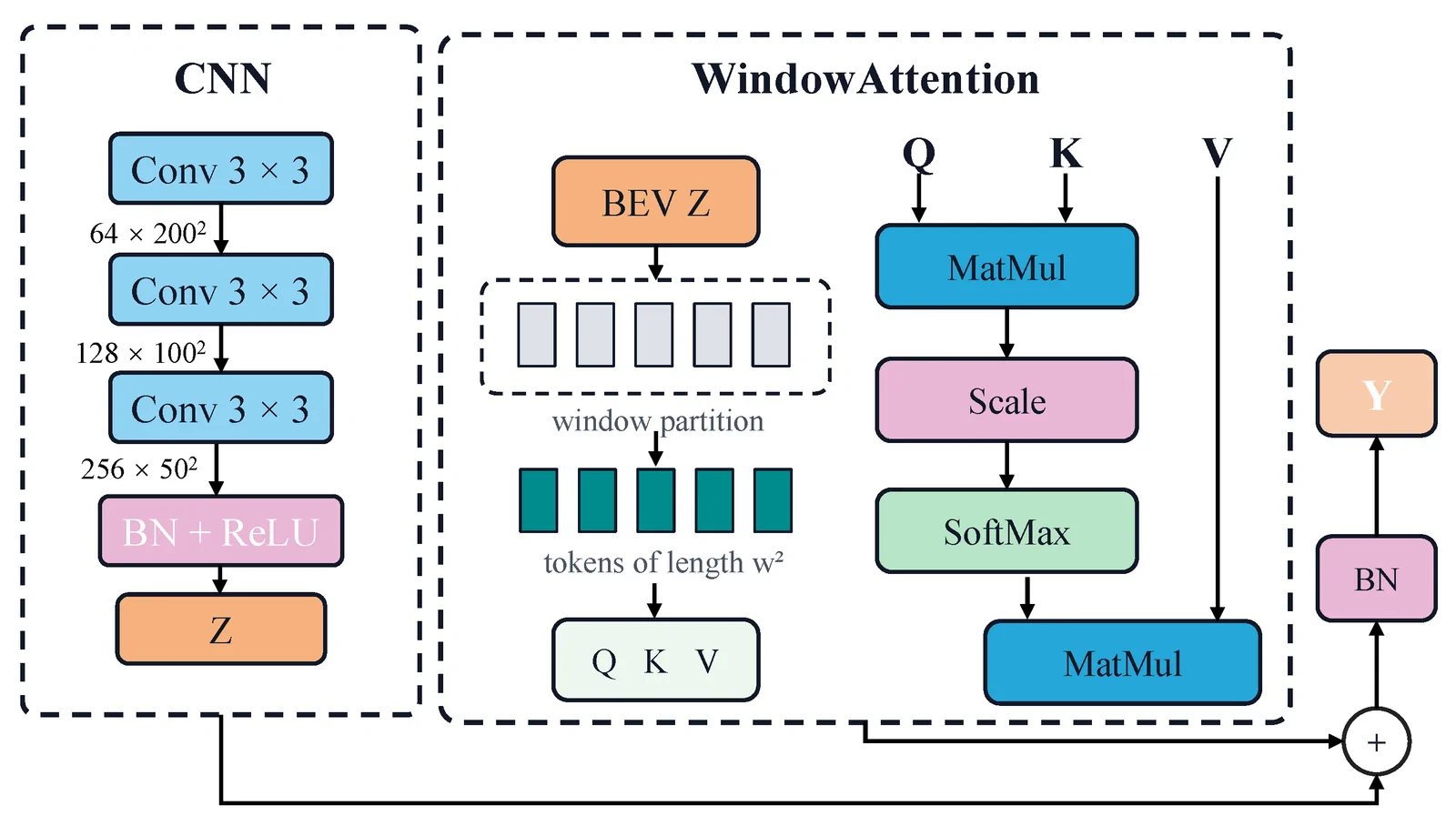
Structure of the lightweight CNN-Transformer backbone. Solid arrows indicate the data flow, dashed boxes delimit the CNN and window-attention modules, different colors distinguish operations and feature tensors, and the circled plus sign denotes residual addition.

To address this issue, this study designs a lightweight CNN-Transformer backbone, whose structure is shown in Figure 5. The core idea of Transformer is to directly model dependencies among features at different positions through attention, thereby overcoming the local receptive-field limitation of convolution. A staged processing strategy is adopted. The first two stages retain convolutional structures to rapidly extract local features on high-resolution feature maps and reduce spatial size. In the third stage, a hybrid CNN-Transformer module is introduced on the deep 50×50 BEV feature map. This avoids direct attention computation on large 400×400 or 200×200 feature maps, controlling computational cost while allowing attention to operate on semantically concentrated deep features.

The input feature X first passes through a 3×3 convolution, batch normalization, and ReLU activation to obtain the locally enhanced feature Z:

(17) $$Z=\mathrm{ReLU}\left(\mathrm{BN}\left({\mathrm{Conv}}_{3\times 3}\left(X\right)\right)\right).$$

The feature Z is then fed into the window-attention module. It partitions the feature map into fixed w×w windows and rearranges each window into a token sequence of length $w^{2}$. For one attention head, linear mappings generate query Q, key K, and value V matrices, and the response is

(18) $$\mathrm{Attention}\left(Q,K,V\right)=\mathrm{softmax}\left(\frac{QK^{T}}{\sqrt{d_{h}}}\right)V.$$

Here, $d_{h}$ is the feature dimension of a single attention head. Compared with global self-attention, window attention computes token relationships only within local windows. Because the third-stage feature-map resolution is already reduced, this operation expands local feature interaction under controlled computational cost.

The output of window attention is linearly projected and then fused with the convolutional feature through a residual connection.

(19) $$Y=\mathrm{BN}\left(Z+\mathrm{WindowAttention}(Z)\right).$$

The residual connection preserves the local geometric responses extracted by the convolutional path and reduces training instability after the attention branch is introduced. The first two stages without Transformer are convolutional feature extraction modules. Therefore, the overall backbone balances convolutional efficiency and the contextual representation ability of attention mechanisms, making it suitable for the current small-scale engineering dataset and real-time perception requirements.

### 3.6. Relative Pose Calculation

**Figure 6:**
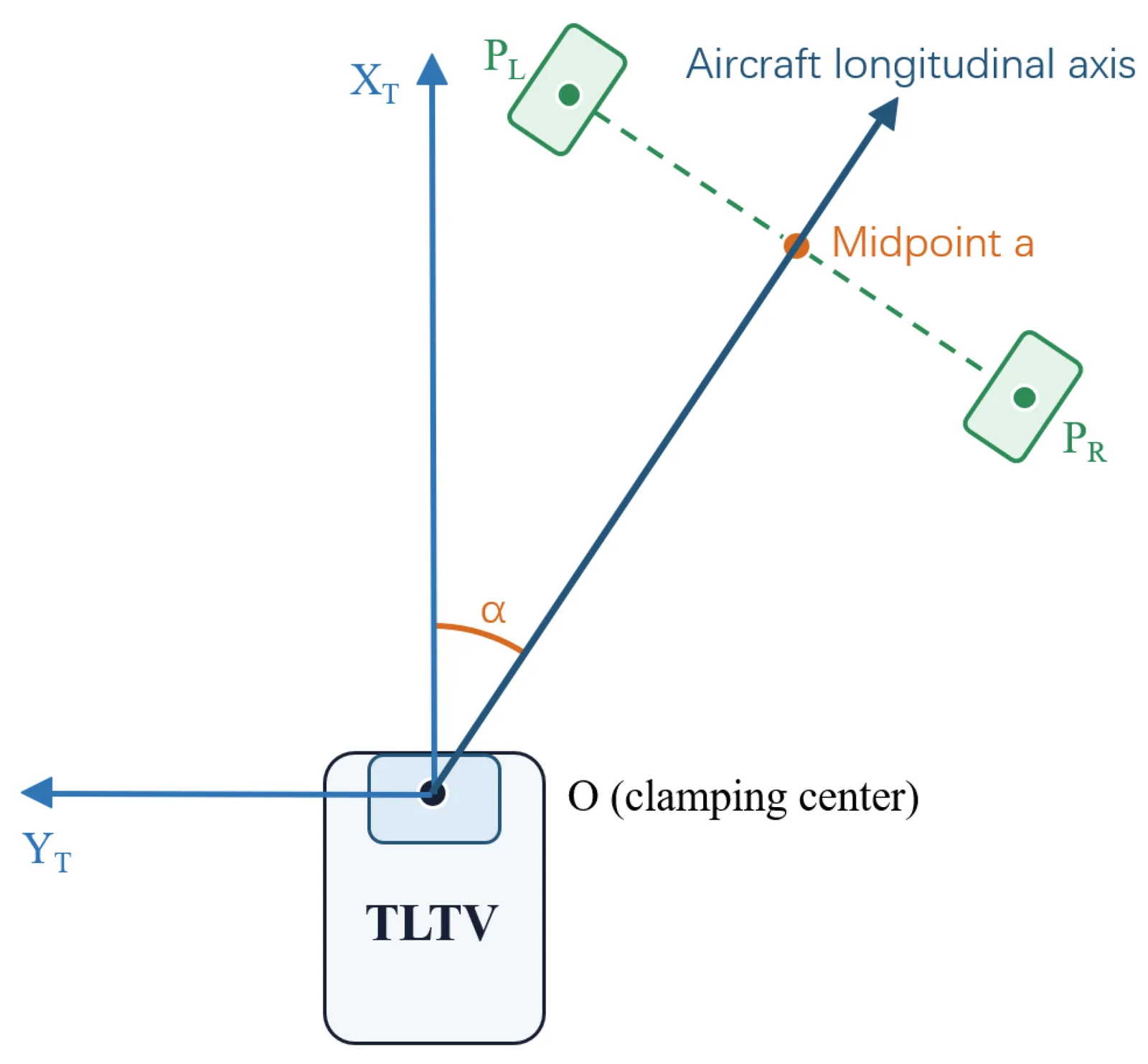
Schematic diagram of relative orientation calculation.

This study uses the TLTV clamping center as the origin of the TLTV coordinate system and defines the longitudinal axis of the towing vehicle as the x-axis in the unified coordinate system. Since the left and right aircraft rear wheels have an approximately symmetric relationship on both sides of the fuselage, the midpoint of the detected left and right rear-wheel centers, hereafter referred to as the rear-wheel midpoint, is treated as the center point of an equivalent rear-wheel axis and used as an observed point on the aircraft fuselage centerline. By combining the clamping center and the rear-wheel midpoint, the planar pose angle of the aircraft relative to the towing vehicle can be obtained, as illustrated in Figure 6.

For a detected rear wheel q∈{L,R}, the network output is written as

(20) $$B_{q}=\left(x_{q},y_{q},z_{q},l_{q},w_{q},h_{q},\theta_{q},s_{q}\right),\;q\in \left\{L,R\right\},$$

where $\left(x_{q},y_{q},z_{q}\right)$ are the box-center coordinates; $\left(l_{q},w_{q},h_{q}\right)$ are its dimensions; $\theta_{q}$ is its heading; $s_{q}$ is its confidence score; and *L* and *R* denote the left and right rear wheels. Only the horizontal center coordinates are used for pose estimation:

(21) $$c_{L}={\left[\begin{array}{cc} x_{L} & y_{L} \end{array}\right]}^{T},\;c_{R}={\left[\begin{array}{cc} x_{R} & y_{R} \end{array}\right]}^{T}.$$

Their midpoint and the clamping-center position are

(22) $$c_{a}=\frac{c_{L}+c_{R}}{2}={\left[\begin{array}{cc} x_{a} & y_{a} \end{array}\right]}^{T},\;c_{0}={\left[\begin{array}{cc} 0 & 0 \end{array}\right]}^{T}.$$

The vector from the clamping center to the rear-wheel midpoint and the unit direction of the TLTV centerline are

(23) $$v_{a}=c_{a}-c_{0}={\left[\begin{array}{cc} x_{a} & y_{a} \end{array}\right]}^{T},\;v_{t}={\left[\begin{array}{cc} 1 & 0 \end{array}\right]}^{T}.$$

The signed relative angle is evaluated without quadrant ambiguity as

(24) $$\alpha =\mathrm{atan2}\left(\det\left(v_{t},v_{a}\right),v_{t}^{T}v_{a}\right)=\mathrm{atan2}\left(y_{a},x_{a}\right).$$

Equation (24) is based on the geometric approximation that the clamping center and the rear-wheel midpoint lie on the aircraft longitudinal axis in the horizontal plane. Let the ideal rear-wheel-midpoint vector be $L{\left[\cos\alpha ,\sin\alpha \right]}^{T}$, where *L* is the longitudinal distance from the clamping center to the rear-wheel midpoint. The geometric offset is denoted by $\delta_{g}$, the midpoint error caused by the detected left- and right-wheel centers is $e_{m}=\left(e_{L}+e_{R}\right)/2$, and the residual translational registration error at the midpoint is $\delta_{t}$. Resolving these quantities along and perpendicular to the aircraft longitudinal axis gives the combined position error

(25) $$\epsilon =\delta_{g}+e_{m}+\delta_{t}={\left[\begin{array}{cc} \epsilon_{\|} & \epsilon_{⊥} \end{array}\right]}^{T}.$$

Here, $\epsilon_{\|}$ denotes the component of the combined position error along the aircraft longitudinal axis, and $\epsilon_{⊥}$ denotes the component perpendicular to that axis in the horizontal plane.

Let $\epsilon_{\psi }=\delta \psi -\delta \beta$ denote the residual planar orientation error, where δψ is the yaw error of the registered point cloud and δβ is the initial alignment error of the TLTV longitudinal axis. The resulting angle error is

(26) $$\Delta \alpha =\hat{\alpha }-\alpha =\epsilon_{\psi }+\mathrm{atan2}\left(\epsilon_{⊥},L+\epsilon_{\|}\right)\approx \epsilon_{\psi }+\frac{\epsilon_{⊥}}{L},\;|\epsilon_{\|}\left|,\right|\epsilon_{⊥}|\ll L.$$

For bounded errors and $L>|\epsilon_{\|}|$, Equation (26) gives

(27) $$\left|\Delta \alpha |\leq \right|\epsilon_{\psi }|+\mathrm{atan}\left(\frac{|\epsilon_{⊥}|}{L-|\epsilon_{\|}|}\right).$$

The first-order result shows that lateral midpoint and translational errors are scaled approximately by 1/L, whereas residual yaw and longitudinal-axis alignment errors enter the estimated angle directly. A purely longitudinal position error has no first-order angular effect when $\epsilon_{⊥}=0$. Length quantities in Equations (22)–(27) use meters and angles use radians internally. Positive α denotes left deflection relative to the TLTV *x*-axis and negative α denotes right deflection.

### 3.7. Bounded Error Sensitivity Analysis

Point clouds acquired from multiple sensors depend on the accuracy of their transformations to a common coordinate frame. Chen et al. examined calibration-related inconsistencies in a multi-camera reconstruction system and corrected overlapping point-cloud regions through clustering and registration [36]. For the dual-LiDAR system considered here, the bounded error model in Equation (27) is used to quantify the influence of residual position and orientation errors on the calculated relative angle.

To provide a reference boundary for the sensitivity analysis, an angular deviation of 1° is selected. When the residual orientation and longitudinal errors are set to zero, the maximum combined lateral error corresponding to an angular boundary τ is

(28) $$|\epsilon_{⊥}{|}_{\max}=L\tan\tau .$$

For the selected boundary, τ=1°. Table 1 gives the angular change caused by a 0.01 m lateral midpoint error and the corresponding lateral-error limit. The three values of *L* correspond to the longitudinal-distance settings used for dataset acquisition. The combined lateral error includes geometric offset, detection-center error, and residual translational registration error at the rear-wheel midpoint.

For L=3.0 m, a 0.01 m combined lateral error produces an angular bias of approximately 0.191°, and the lateral-error limit corresponding to the 1° boundary is approximately 0.052 m when the other error terms are zero. As *L* increases to 4.5 and 6.0 m, the angular bias produced by the same lateral error decreases to 0.127° and 0.095°, while the corresponding lateral-error limits increase to 0.079 m and 0.105 m, respectively. A residual planar orientation error enters the first-order expression directly, whereas a purely longitudinal error has no first-order angular effect.

## 4. Experimental Analysis

### 4.1. Experimental Conditions and Dataset

Because true aircraft point-cloud data cannot currently be collected under available site conditions and no public point-cloud dataset exists for oversteering perception of towbarless towing vehicles, this study uses a simulated experimental platform for model validation. The experimental objects include a TLTV simulation platform, an inflatable aircraft model, and tires, which reproduce relative pose changes between the towing vehicle and the aircraft as well as changes in rear-wheel visibility. The detailed components are listed in Table 2. This setup provides a stable data source for dual-LiDAR rear-wheel perception and relative pose calculation while maintaining experimental repeatability.

**Figure 7:**
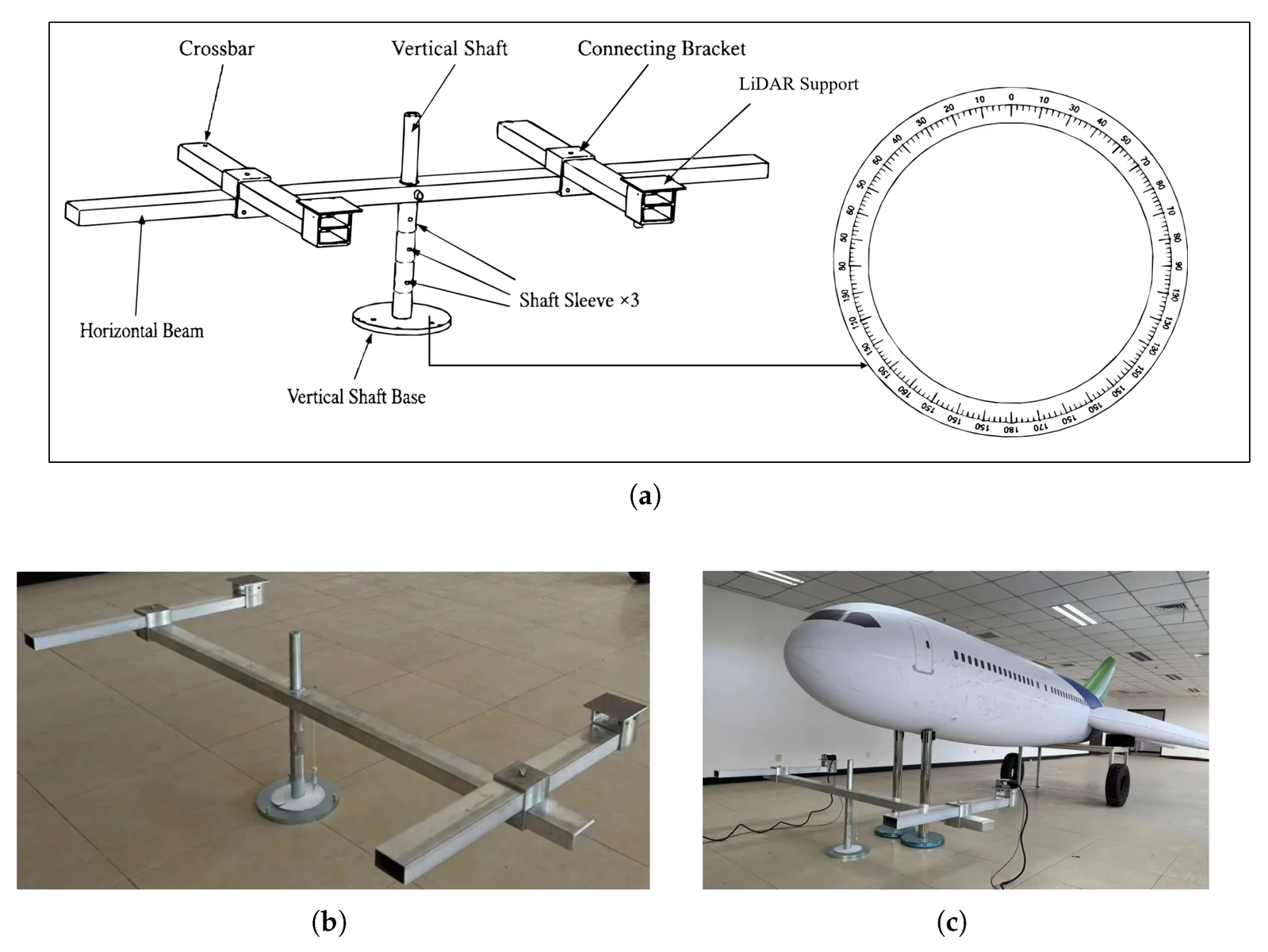
TLTV simulation platform and data-acquisition setup: (**a**) SolidWorks design; (**b**) physical steering simulation platform; (**c**) data-acquisition and simulation-validation scene.

A survey of several aircraft tow tractor models from one manufacturer showed that their widths range from approximately 2.0 to 2.3 m, while the available LiDAR mounting heights range from approximately 0.3 to 0.8 m. Based on these dimensions, a three-dimensional model was developed using SolidWorks, as shown in Figure 7a. The physical tow-tractor steering simulation platform is shown in Figure 7b. The crossbeam can rotate relative to the central vertical shaft, and a transverse support arm is symmetrically connected to each end of the crossbeam. A mounting base for a solid-state LiDAR sensor is fixed to the same side of each support arm. This configuration simulates the relative steering motion between the aircraft tow tractor and the aircraft during ground towing operations. A suspension hole is provided near the midpoint of the crossbeam, from which a pointer is suspended over an angular scale. The relative rotation angle between the rotating device and the aircraft model can be determined from the pointer reading on the graduated dial.

The dataset was constructed using the experimental platform and target objects described above. The point clouds were acquired using physical LiDAR sensors and fused according to the point-cloud registration procedure. Three front-to-rear wheel spacings of 3, 4.5, and 6 m were used, with the main-wheel track fixed at 2 m. The complete inflatable aircraft model was used for the 3 m setting, as shown in Figure 7c. Owing to the dimensions of the inflatable model, the 4.5 and 6 m settings retained only the tire targets to provide observations of isolated-wheel point-cloud characteristics at different longitudinal distances. Because both longitudinal distance and scene composition varied among the three settings, they were treated as dataset acquisition configurations rather than as a controlled aircraft-wheelbase generalization experiment, and their results were not used to infer generalization across aircraft geometries.

**Figure 8:**
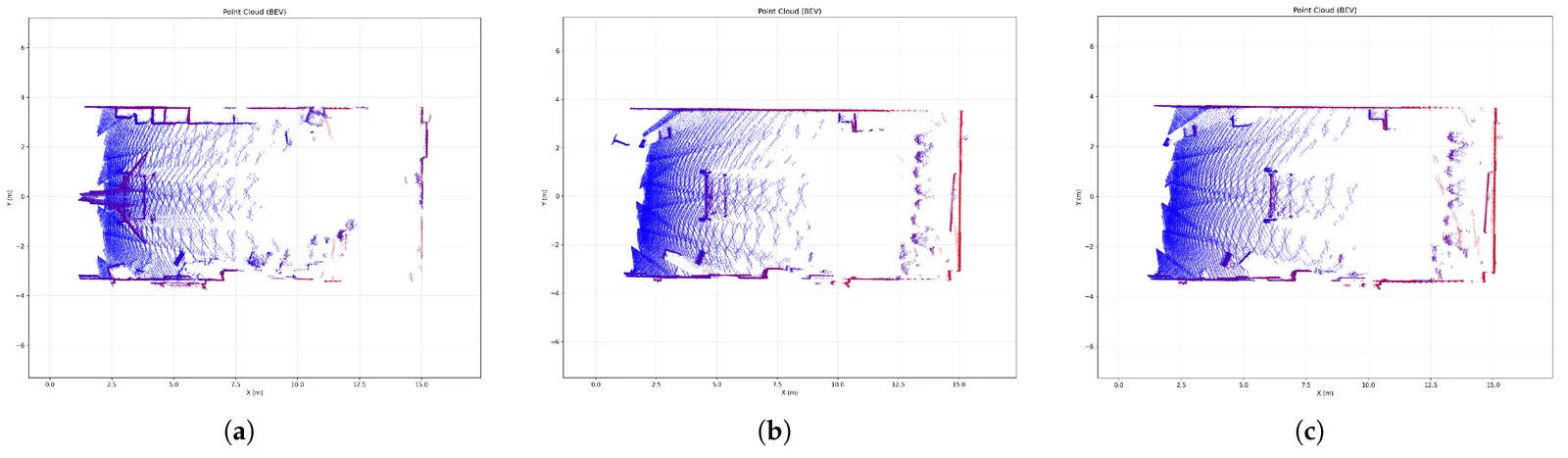
Representative fused point clouds at different front-to-rear wheel spacings: (**a**) 3 m; (**b**) 4.5 m; (**c**) 6 m.

Left-turn and right-turn point-cloud sequences were collected separately over a towing-angle range from −40° to +40° by rotating the towing-vehicle simulation platform. Because the LiDAR sensors acquired data continuously at 10 Hz, adjacent frames had strong temporal correlation, and individual frames were not assigned randomly to the training and validation sets. Within each temporally ordered acquisition sequence, eight consecutive frames were assigned to the training set, followed by one excluded transition frame, two consecutive validation frames, and a second excluded transition frame. This 8–1–2–1 allocation pattern was repeated to the end of each sequence. Applying this rule across the acquisition sequences produced 587 frames, comprising 399 training frames, 94 validation frames, and 94 excluded transition frames. Both the training and validation sets contained all three longitudinal-distance settings and both turning directions, and the same fixed split was used for all models. No independent test set was created, and the detection results reported in this paper were obtained on the fixed validation set. The point-cloud frames retained for training and validation were annotated using LabelCloud. Each frame contains two oriented 3D bounding boxes corresponding to the left and right rear wheels, and both boxes belong to the single rear-wheel category. After the initial annotation, all bounding boxes were reviewed frame by frame in LabelCloud to verify target completeness, class labels, box positions, dimensions, and orientations. Inconsistent annotations were corrected before the annotations were stored in KITTI format. Representative fused point clouds for the three front-to-rear wheel spacings are presented in Figure 8.

During training, each point cloud and its corresponding 3D bounding boxes were jointly augmented by a random rotation within [−0.7854,0.7854] rad, scaling within [0.90,1.10], zero-mean random translation with standard deviations of [0.5,0.5,0.2] m along the three axes, and horizontal BEV flipping with a probability of 0.5. Vertical BEV flipping was disabled because the longitudinal detection range, x∈[0,40] m, is not symmetric about the origin. After these geometric transformations, point- and object-range filtering was applied to the transformed coordinates, followed by point shuffling and input packing. The validation pipeline did not use stochastic geometric augmentation.

The principal ablation and representative-detector comparisons were repeated using random seeds 1, 42, and 2026. All models used the same fixed training/validation split, preprocessing and augmentation pipeline, evaluation metrics, and experimental hardware. The reported accuracy metrics are the mean and sample standard deviation over the three runs. Parameter counts and FPS were measured in the same inference environment. The bin-number, window-size, and backbone-control experiments changed one setting at a time and are reported separately as controlled single-run results.

**Figure 9:**
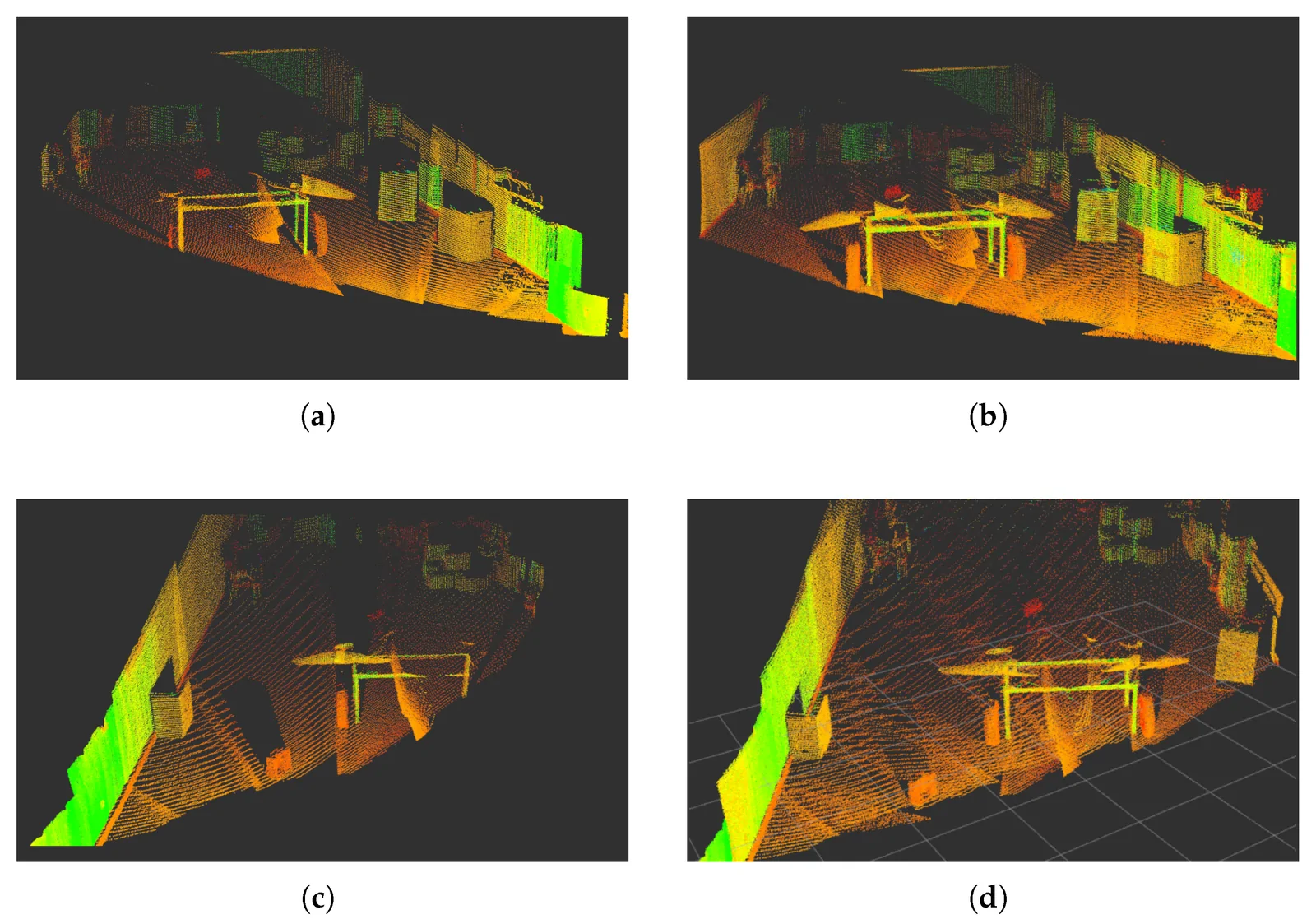
Qualitative comparison of point-cloud coverage at two large steering angles: (**a**,**c**) single-LiDAR observations; (**b**,**d**) the corresponding registered dual-LiDAR point clouds.

To illustrate the complementary observations provided by the two sensors, Figure 9 compares single-LiDAR point clouds with the corresponding registered dual-LiDAR point clouds at large steering angles. In the single-sensor views, parts of the rear-wheel region are sparsely sampled or occluded because of the relative orientation between the towing platform and the aircraft model. After registration and fusion, returns acquired from the opposite viewpoint supplement these regions and provide more complete point-cloud coverage of the rear wheels. The fused dual-LiDAR point clouds are used as the input to the subsequent detection and pose-estimation processes.

### 4.2. Dual-LiDAR Temporal Pairing and Spatial Registration

To characterize the timestamp differences produced by the software pairing, 200 paired point-cloud messages were evaluated. The approximate timestamp synchronizer used a queue size of 2 and a tolerance of 0.1 s. Defining the timestamp difference as $\Delta t=t_{1}-t_{2}$, where $t_{1}$ and $t_{2}$ are the message-header timestamps of LiDAR 1 and LiDAR 2, respectively, the signed mean offset was −34.59 ms with a standard deviation of 6.79 ms. The absolute timestamp differences are summarized in Table 3. The negative signed mean indicates that the timestamps of LiDAR 1 generally preceded those of LiDAR 2.

The spatial alignment obtained using the fixed extrinsic transformation was evaluated on 20 dual-LiDAR point-cloud pairs. The same transformation used for dataset construction was applied to each pair, after which nearest-neighbor Euclidean and point-to-plane distances were calculated for 213,964 valid correspondences. Table 4 reports the post-registration geometric residuals. Across the 20 point-cloud pairs, the fixed extrinsic transformation resulted in a mean point-to-plane residual of 0.0403 m, an RMSE of 0.0814 m, and a median residual of 0.0168 m. The corresponding 95th-percentile value was 0.1720 m.

### 4.3. Evaluation Metrics

The evaluation metrics used in this study include 3D mAP at IoU thresholds of 0.25 and 0.5, Precision, Recall, and F1-score. For a fixed IoU threshold τ, the AP for class *c* is the area under its interpolated Precision–Recall curve:

(29) $${\mathrm{AP}}_{c}\left(\tau \right)=\int_{0}^{1}P_{c}\left(R;\tau \right)\;dR.$$

(30) $$\mathrm{mAP}\left(\tau \right)=\frac{1}{C}\sum_{c=1}^{C}{\mathrm{AP}}_{c}\left(\tau \right).$$

Here, *C* is the number of classes and $P_{c}\left(R;\tau \right)$ denotes the interpolated Precision of class *c* at Recall *R* under threshold τ. Since this is a single-class task, C=1 and mAP equals the AP of the wheel class.

To supplement the limited interpretability of AP on a single-class small-scale dataset, Precision, Recall, and F1-score are further used to describe detection stability. Under unified IoU threshold, confidence threshold, and NMS settings, they are defined as follows.

(31) $$\mathrm{Precision}=\frac{TP}{TP+FP}.$$

(32) $$\mathrm{Recall}=\frac{TP}{TP+FN}.$$

(33) $$F_{1}=2\;\frac{\mathrm{Precision}\cdot \mathrm{Recall}}{\mathrm{Precision}+\mathrm{Recall}}.$$

Here, TP denotes the number of correctly detected rear-wheel targets, FP denotes the number of false positives, and FN denotes the number of missed detections. Precision reflects the reliability of detection results, Recall reflects the completeness of target detection, and F1-score summarizes the balance between false positives and missed detections.

### 4.4. Ablation and Hyperparameter Analysis

The number of vertical bins *B* and the local-window size *w* were selected before the principal ablation experiments. Each parameter was varied in a controlled single-run comparison while the remaining settings were held fixed. The results are given in Table 5.

For VDE, B=8 produces the highest mAP@0.5 and F1-score among the three settings, whereas B=16 gives a slightly higher mAP@0.25. The B=8 setting is retained because it provides the most balanced result under the stricter IoU threshold. For LTB, w=5 gives the highest mAP@0.5 and F1-score. Increasing the window to 10 raises mAP@0.25 but reduces mAP@0.5, Recall, and F1-score, indicating that the larger local region does not improve the stricter localization criterion in this experiment.

Based on these controlled results, the principal ablation uses B=8 for vertical density enhanced encoding and w=5 for local window attention. Four PointPillars-based configurations are compared: the original network as the Baseline; the Baseline with vertical density enhanced encoding using B=8, denoted as +VDE; the Baseline with the lightweight CNN-Transformer BEV backbone using w=5, denoted as +LTB; and the Full model containing both modifications with B=8 and w=5. The results are given in Table 6.

Relative to the Baseline, +VDE and +LTB increase the mean mAP@0.5 by 0.0473 and 0.0370, respectively. These differences are interpreted together with the observed run-to-run variability rather than as isolated point estimates. The Full model reaches an mAP@0.5 of 0.8788±0.0161, which is 0.0850 higher than the Baseline, and increases the mean Precision, Recall, and F1-score to 0.9689, 0.8972, and 0.9250, respectively.

The Full model contains 4.1069 M parameters, 0.7157 M fewer than the Baseline, while its inference rate decreases from 39.2728 to 38.0732 FPS and remains above the 10 Hz LiDAR acquisition rate. The joint configuration has the highest mAP@0.5, Precision, Recall, and F1-score among the four PointPillars-based configurations, whereas the individual module increments are smaller and more variable across seeds.

The LTB configuration changes both the convolutional depth and the use of local attention. Table 7 separates these factors by comparing the original [3,5,5] CNN, a reduced [2,3,3] CNN without attention, and the [2,3,3] LTB with local attention.

The reduced [2,3,3] CNN without attention yields an mAP@0.5 of 0.8205, compared with 0.8501 for the original [3,5,5] CNN. At the same [2,3,3] depth, local attention increases mAP@0.5 to 0.8535 and Recall to 0.8777. This control separates the effect of local attention from that of network depth and supports the use of LTB as a lightweight backbone replacement.

### 4.5. Comparison with Representative Detectors

After selecting B=8 and w=5 and evaluating the component contributions, the resulting Full configuration was compared with the original PointPillars baseline and the representative CenterPoint and PillarNet detectors. Table 8 reports the mean and sample standard deviation from three runs for each detector.

PillarNet obtains the highest mAP@0.5 at 0.9416±0.0092, while CenterPoint obtains the highest mAP@0.25 and Recall. The Full model reaches the highest Precision, 0.9689±0.0059, but its mAP values remain below those of CenterPoint and PillarNet. Its parameter count is 4.1069 M, compared with 5.2186 M for CenterPoint and 13.5820 M for PillarNet. The comparison therefore places the proposed model as a compact task-adapted detector rather than as a uniformly superior general-purpose architecture.

### 4.6. Towing Angle Verification

**Figure 10:**
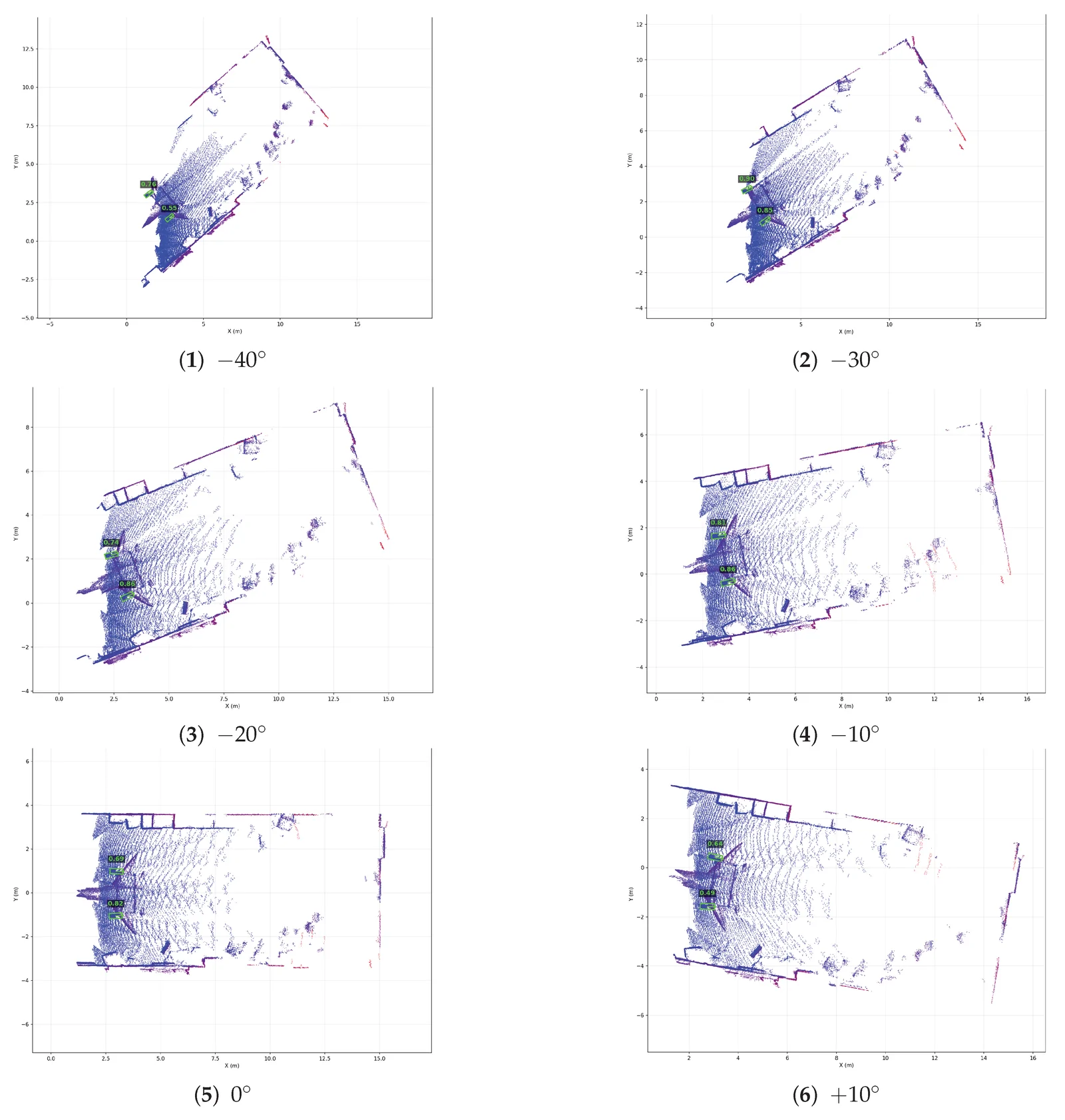

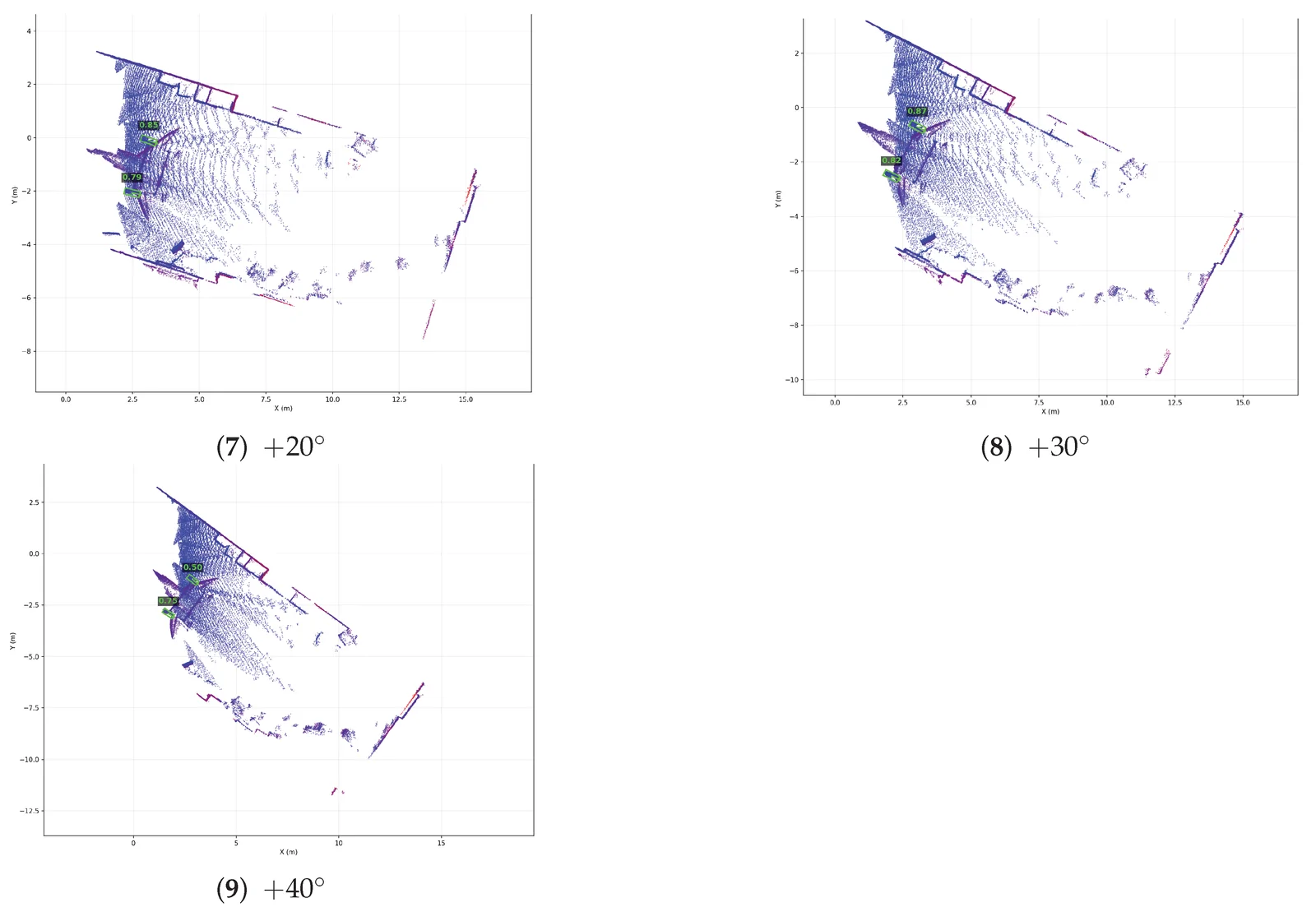
Representative BEV detection results at the nine reference towing angles (part 1 of 2). Panels (**1**)–(**4**) correspond to reference angles from −40° to −10°. Blue and red points represent the registered point clouds from the two LiDAR sensors, respectively; green boxes indicate the detected rear-wheel targets, with the associated confidence scores. Representative BEV detection results at the nine reference towing angles (continued; part 2 of 2). Panels (**5**)–(**9**) correspond to reference angles from 0° to +40°. Blue and red points represent the registered point clouds from the two LiDAR sensors, respectively; green boxes indicate the detected rear-wheel targets, with the associated confidence scores.

The experiments were conducted using the setup shown in Figure 7c, with a main-wheel track of 2 m and a front-to-rear wheel spacing of 3 m. The relative pose was represented by the steering angle. The estimated angle was calculated from the centers of the left and right rear-wheel detection boxes obtained after registration and fusion of the dual-LiDAR point clouds, whereas the reference angle was determined from the graduated dial of the tow-tractor simulation platform. Nine reference steering angles were set at 10° intervals from −40° to +40°. Ten point-cloud frames were evaluated at each reference angle, yielding 90 frame-level observations. The platform remained at the prescribed position during each ten-frame sequence, so the reported standard deviations describe short-term frame-to-frame variation under static conditions. The steering direction determined the sign of the angle, with left turns defined as positive and right turns as negative. Representative BEV point clouds and rear-wheel detection results are shown in Figure 10, while the repeated-measurement statistics are presented in Table 9.

As shown in Figure 10, the paired rear-wheel targets are detected at all nine reference towing angles, and valid two-wheel detections were obtained in all 90 evaluated frames. From panels (1) to (9), the orientation of the registered scene point cloud changes consistently with the imposed rotation, while the detection boxes remain aligned with the corresponding rear-wheel point clusters. Figure 10 provides representative qualitative results, whereas the frame-level angle statistics are reported in Table 9. Because the coordinate limits are adjusted independently according to the visible extent of each scene, object dimensions and displacements should not be compared directly across panels.

Across the 90 frame-level observations in Table 9, the overall MAE is 1.11°, the RMSE is 1.22°, and the maximum absolute error is 2.17°. The towing-angle estimation errors remain within the allowable engineering range.

The within-angle standard deviations range from 0.15° to 0.41°, describing the short-term variation of the angle output while the platform remains at a fixed position.

### 4.7. Failure-Case Analysis

Detection failures were examined using the seed-42 checkpoint of the Full model on the fixed validation split. The validation set contains 94 point-cloud frames and 188 ground-truth rear-wheel boxes. Predictions with confidence scores of at least 0.3 were matched one-to-one to ground-truth boxes of the same class using a 3D IoU threshold of 0.5. Among 174 retained predictions, 165 were true positives and 9 were false positives, while 23 ground-truth boxes remained unmatched. At least one false positive or false negative occurred in 19 of the 94 frames. The resulting Precision, Recall, and F1-score were 0.9483, 0.8777, and 0.9116, respectively. These single-checkpoint results are used to characterize the observed failure modes and are separate from the three-seed aggregate results reported in Table 6 and Table 8.

**Figure 11:**
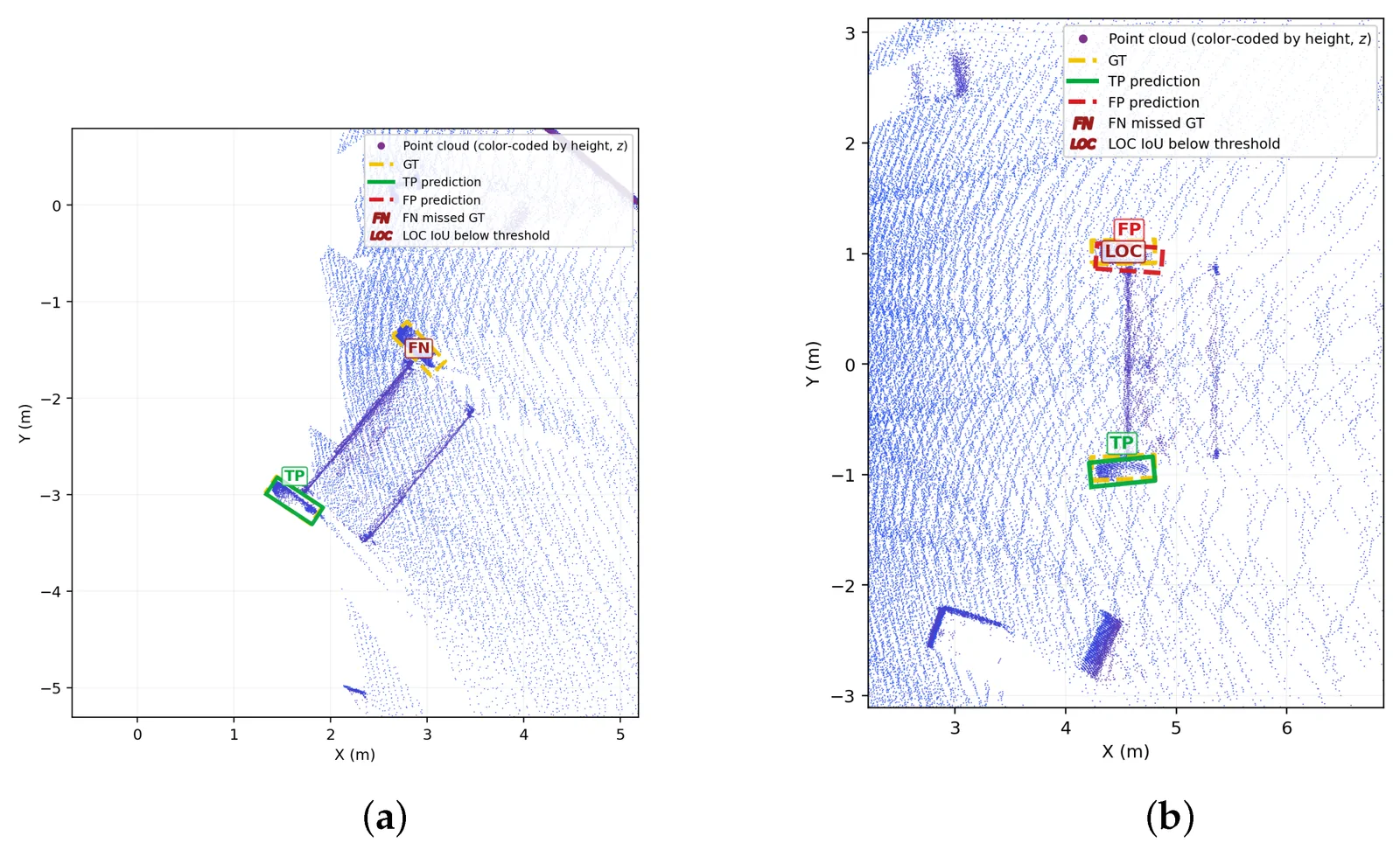
Representative detection failures of the Full model using a confidence threshold of 0.3 and a 3D IoU threshold of 0.5: (**a**) a genuine missed rear-wheel target with no nearby prediction; (**b**) a localization failure in which a nearby prediction does not reach the IoU threshold. The point-cloud colors encode the height coordinate z. Yellow dashed boxes denote ground truth, green solid boxes denote true-positive predictions, and red dashed boxes denote unmatched predictions. The FN label is used only when no nearby prediction exists. LOC denotes a nearby prediction below the IoU threshold; under the formal matching rule, this prediction contributes one FP and the corresponding unmatched ground-truth box contributes one FN.

Figure 11a shows a frame in which one rear wheel is detected while the other ground-truth target has no adjacent predicted box. Figure 11b shows a different outcome: the second prediction is located near the corresponding target but its 3D overlap does not satisfy the 0.5 threshold. The two examples distinguish a complete missed detection from insufficient box localization. They are presented as observed diagnostic cases and do not establish a specific environmental or sensor-related cause for either failure.

## 5. Conclusions

This study proposes a point-cloud perception method that integrates dual-LiDAR acquisition, rear-wheel target detection, and relative pose calculation for oversteering-risk perception during ground transfer with a towbarless towing vehicle. Considering nose-landing-gear occlusion during towing and the paired distribution of aircraft rear wheels, the left and right rear wheels are used as detection targets. The dual-LiDAR point clouds are registered into a unified coordinate system to provide spatial information for subsequent towing-angle estimation.

For the detection model, PointPillars is used as the baseline, and vertical density enhanced encoding and a lightweight CNN-Transformer BEV backbone are introduced. Across three random seeds, the Full model obtains an mAP@0.5 of 0.8788±0.0161, compared with 0.7938±0.0411 for the Baseline. Its Precision, Recall, and F1-score are 0.9689±0.0059, 0.8972±0.0134, and 0.9250±0.0092, respectively. The model contains 4.1069 M parameters and runs at 38.0732 FPS. CenterPoint and PillarNet obtain higher mAP values in the representative-detector comparison, while the proposed model uses fewer parameters. The results position the model as a compact task-specific adaptation that improves the PointPillars baseline while satisfying the real-time requirement of the 10 Hz LiDAR system.

The towing-angle verification results show that calculating relative pose from rear-wheel detection-box centers is feasible under the current controlled setup. Across 90 frame-level observations at nine reference angles from −40° to +40°, the MAE, RMSE, and maximum absolute error are 1.11°, 1.22°, and 2.17°, respectively, and the towing-angle estimation errors remain within the allowable engineering range.

This study is still limited by the experimental platform, dataset scale, and validation scenarios. The current data are collected using a TLTV simulation platform and a simulated aircraft model, and they do not yet cover complex apron environments, different aircraft sizes, or long-term sensor installation errors in real aircraft towing. Future work will expand the dataset under real or more realistic aircraft towing conditions and integrate the perception results with oversteering-threshold judgment, warning strategies, and towing-control systems for joint validation. 

## Figures and Tables

**Table 1:** Bounded lateral-error sensitivity of the relative-pose calculation.

*L* (m)	Angular Bias for 0.01 m Lateral Error (°)	Ltan1° (m)
3.0	0.191	0.052
4.5	0.127	0.079
6.0	0.095	0.105

**Table 2:** Composition of the experimental platform.

Component	Main Parameters
Advantech industrial computer	i5-10400T; RTX 4060; 32 GB; 1 TB; Ubuntu 22.04
RoboSense M1 solid-state LiDAR	10 Hz; vertical field of view 20°; horizontal field of view 120°
Dual-LiDAR installation parameters	spacing 2 m; height 0.47 m; midpoint as the clamping center
TLTV simulation platform	/
Inflatable aircraft model	fuselage length approximately 6 m; fuselage diameter approximately 0.7 m; side wing length approximately 2 m
Tire	diameter 0.58 m; tread width 0.22 m

**Table 3:** ROS-message timestamp differences for dual-LiDAR pairing.

Metric	Timestamp Difference (ms)
Signed mean	−34.59
Standard deviation	6.79
Median absolute difference	33.36
95th-percentile absolute difference	44.20
99th-percentile absolute difference	61.99
Maximum absolute difference	82.21

**Table 4:** Post-registration geometric residuals obtained using the fixed extrinsic transformation.

Distance Measure	Mean (m)	RMSE (m)	Median (m)	P95 (m)
Euclidean	0.1150	0.1733	0.0614	0.4128
Point-to-plane	0.0403	0.0814	0.0168	0.1720

**Table 5:** Sensitivity to the number of vertical bins and local-window size.

Setting	Value	mAP@0.25	mAP@0.5	Precision	Recall	F1-Score
*B*	4	0.8989	0.8367	0.9586	0.8617	0.9076
*B*	8	0.9202	0.8595	0.9538	0.8777	0.9141
*B*	16	0.9255	0.8502	0.9483	0.8777	0.9116
*w*	2	0.9255	0.8318	0.9310	0.8617	0.8950
*w*	5	0.9202	0.8466	0.9364	0.8617	0.8975
*w*	10	0.9522	0.7665	0.8766	0.8166	0.8455

**Table 6:** Three-seed ablation results, reported as mean ± sample standard deviation.

Model	mAP@0.25	mAP@0.5	Precision	Recall	F1-Score	Params (M)	FPS
Baseline	0.9306±0.0381	0.7938±0.0411	0.8966±0.0309	0.8027±0.0673	0.8467±0.0511	4.8226	39.2728
+VDE	0.9417±0.0299	0.8411±0.0172	0.9278±0.0217	0.8682±0.0306	0.8969±0.0247	4.8272	38.1853
+LTB	0.9196±0.0170	0.8308±0.0198	0.9217±0.0147	0.8387±0.0430	0.8780±0.0301	4.1022	39.3291
Full	0.9398±0.0110	0.8788±0.0161	0.9689±0.0059	0.8972±0.0134	0.9250±0.0092	4.1069	38.0732

**Table 7:** Controlled comparison of CNN depth and local attention.

Backbone	CNN Depth	Attention	mAP@0.25	mAP@0.5	Precision	Recall	F1-Score
Original CNN	[3,5,5]	No	0.9255	0.8501	0.9425	0.8723	0.9061
Reduced CNN	[2,3,3]	No	0.9043	0.8205	0.9350	0.8460	0.8880
LTB	[2,3,3]	Yes	0.9362	0.8535	0.9375	0.8777	0.9066

**Table 8:** Comparison with representative 3D detectors over three random seeds.

Model	mAP@0.25	mAP@0.5	Precision	Recall	F1-Score	Params (M)	FPS
PointPillars	0.9306±0.0381	0.7938±0.0411	0.8966±0.0309	0.8027±0.0673	0.8467±0.0511	4.8226	39.2728
CenterPoint	0.9917±0.0048	0.9161±0.0234	0.9468±0.0192	0.9468±0.0192	0.9434±0.0155	5.2186	16.6283
PillarNet	0.9644±0.0063	0.9416±0.0092	0.9541±0.0089	0.9298±0.0099	0.9418±0.0094	13.5820	45.9200
Full	0.9398±0.0110	0.8788±0.0161	0.9689±0.0059	0.8972±0.0134	0.9250±0.0092	4.1069	38.0732

**Table 9:** Repeated static-frame towing-angle verification results.

Reference Angle (°)	*n*	Estimated Angle (°), Mean ± SD	MAE (°)	RMSE (°)	Max. Error (°)
−40	10	−40.79±0.16	0.79	0.81	1.04
−30	10	−30.00±0.24	0.17	0.23	0.52
−20	10	−21.18±0.15	1.18	1.18	1.38
−10	10	−11.25±0.20	1.25	1.27	1.63
0	10	+1.04±0.34	1.04	1.09	1.63
+10	10	+9.18±0.41	0.87	0.90	1.19
+20	10	+18.70±0.22	1.30	1.32	1.75
+30	10	+28.49±0.33	1.51	1.54	2.03
+40	10	+38.11±0.18	1.89	1.90	2.17
Overall	90	–	1.11	1.22	2.17

## Data Availability

The data presented in this study are available from the corresponding author upon reasonable request.
